# Methodological Challenges in International Comparisons of Perinatal Mortality

**DOI:** 10.1007/s40471-017-0101-4

**Published:** 2017-04-17

**Authors:** K. S. Joseph, Neda Razaz, Giulia M. Muraca, Sarka Lisonkova

**Affiliations:** 1 Department of Obstetrics and Gynaecology and the School of Population and Public Health, University of British Columbia and the Children’s and Women’s Hospital and Health Centre of British Columbia, Room C403, Women’s Hospital of British Columbia, 4500 Oak Street, Vancouver, BC V6H 3N1 Canada; 2 Reproductive Epidemiology Research Unit, Department of Medicine, Karolinska Institutet, Stockholm, Sweden

**Keywords:** Perinatal mortality, International, Methods, Gestational age, Birth weight

## Abstract

**Purpose of Review:**

Several prestigious agencies routinely rank countries based on crude perinatal and infant mortality rates, while more recently, international neonatal networks have begun comparing neonatal mortality and morbidity rates among very preterm and very low-birth-weight infants. We discuss the methodologic challenges that compromise such comparisons and potential remedies.

**Recent Findings:**

Crude perinatal mortality rates are biased by international variations in birth registration, especially at the borderline of viability. Such bias is demonstrated by significant differences in crude versus birth weight- and gestational age-specific comparisons of perinatal mortality. Comparisons of neonatal mortality among very preterm and very low-birth-weight infants are plagued by incorrect denominators, and this leads to paradoxical findings.

**Summary:**

A lack of standardization with regard to birth registration and inadequate appreciation of the methods for calculating gestational age-specific mortality rates are responsible for biasing international comparisons of perinatal mortality.

## Introduction

International comparisons of perinatal and infant mortality are appealing for various sociological reasons and can serve to spur improvements in a country’s health system performance. Towards this end, several prestigious institutions such as the UNICEF, the World Bank, and the Organization for Economic Cooperation and Development (OECD) publish annual rankings of countries by rates of stillbirth, infant mortality, and child mortality under 5 years of age [1–3]. These reports create newspaper and other media headlines worldwide and lead to colorful commentaries and political rhetoric by vested interests. Ignored in this rush to judgement is the validity of such information and the methodologic challenges that compromise international comparisons of fetal and infant mortality.

Problems with international comparisons of perinatal mortality are not restricted to routine reports published by the UNICEF, the World Bank, the OECD, and related institutions. Serious methodologic issues also plague international comparisons of neonatal mortality that have appeared in scientific medical journals [4–7]. These latter research papers were based on data from country-wide networks of neonatal intensive care units and provided contrasts of neonatal mortality and severe neonatal morbidity among infants in the very preterm birth or very low-birth-weight range. Although the challenges that invalidate such birth weight- and gestational age-specific comparisons of neonatal mortality/morbidity are more esoteric than those affecting crude comparisons of overall fetal and infant mortality, there is a substantial literature documenting the methodologic problems that beset comparisons within such restricted subpopulations [8–18]. In this paper, we discuss the substantive and methodologic issues that bias and compromise international comparisons of perinatal mortality.

## Problems with Crude Comparisons of Stillbirth and Neonatal Death

There is substantial variation in stillbirth registration criteria internationally, and this makes crude comparisons of national stillbirth rates less than meaningful. Most of the variation results from differences in stillbirth registration at the borderline of viability, and thus, rankings of countries based on crude stillbirth rates tend to be substantially different from ranking of countries based on stillbirth rates among births with a birth weight ≥1000 g (or a gestational age ≥28 weeks). Table 1 shows information on stillbirths from selected high-income countries taken from a study published in the *BMJ* in 2012 [19]; Sweden ranked first and Australia ranked 12th based on crude stillbirth rates, whereas these countries placed 4th and 5th, respectively, in comparisons of stillbirth rates among births ≥1000-g birth weight (Table 1). The crude stillbirth rate in Sweden was less than half the stillbirth rate in Australia (rate ratio [RR] 0.42, 95% confidence interval [CI] 0.37–0.47]), whereas the stillbirth rate among births ≥1000-g birth weight in Sweden was not significantly different from the same stillbirth rate in Australia (RR 0.96, 95% CI 0.84–1.10).

**Table 1:** Crude and birth weight-specific stillbirth and neonatal mortality rates from selected high-income countries, 2004

Country	Crude mortality			Mortality among births ≥1000 g		
	Rate per 1000 total/live births	Rank	Rate ratio (95% CI)	Rate per 1000 total/live births	Rank	Rate ratio (95% CI)
Stillbirths						
Sweden	3.15	1	0.42 (0.37–0.47)	2.99	4	0.96 (0.84–1.10)
Finland	3.29	2	0.44 (0.38–0.51)	1.97	1	0.63 (0.52–0.77)
Germany	3.48	3	0.47 (0.44–0.50)	2.40	2	0.77 (0.71–0.84)
Czech Republic	3.95	4	0.53 (0.47–0.59)	2.56	3	0.82 (0.71–0.95)
Estonia	4.48	5	0.60 (0.47–0.77)	3.37	7	1.08 (0.81–1.45)
Poland	4.86	6	0.65 (0.61–0.70)	3.55	8	1.14 (1.05–1.25)
England and Wales	5.73	7	0.77 (0.73–0.81)	3.84	11	1.23 (1.14–1.34)
USA	6.53	8	0.88 (0.84–0.92)	3.62	9	1.16 (1.08–1.25)
Latvia	6.69	9	0.90 (0.75–1.07)	4.71	12	1.51 (1.22–1.87)
Netherlands	6.98	10	0.94 (0.87–1.00)	3.81	10	1.23 (1.11–1.36)
Canada	7.11	11	0.95 (0.88–1.03)	3.35	6	1.08 (0.96–1.21)
Australia (reference)	7.46	12	1.00 (−)	3.11	5	1.00 (−)
Neonatal deaths						
Sweden	2.10	1	0.47 (0.41–0.54)	1.56	4	0.97 (0.83–1.14)
Czech Republic	2.29	2	0.51 (0.45–0.58)	1.12	1	0.70 (0.58–0.84)
Finland	2.45	3	0.55 (0.46–0.65)	1.31	2	0.81 (0.65–1.02)
Germany	2.93	4	0.65 (0.62–0.69)	1.49	3	0.92 (0.87–0.99)
England and Wales	3.42	5	0.76 (0.73–0.80)	1.77	7	1.10 (1.03–1.17)
Netherlands	3.49	6	0.78 (0.72–0.84)	1.96	8	1.22 (1.10–1.36)
Denmark	3.56	7	0.80 (0.70–0.91)	2.09	9	1.30 (1.10–1.54)
Canada	3.75	8	0.84 (0.78–0.90)	1.63	6	1.01 (0.91–1.13)
Estonia	4.22	9	0.94 (0.73–1.22)	2.51	10	1.56 (1.12–2.17)
USA (reference)	4.47	10	1.00 (−)	1.61	5	1.00 (−)
Poland	4.85	11	1.08 (1.03–1.14)	2.93	11	1.82 (1.70–1.94)
Latvia	5.70	12	1.27 (1.06–1.53)	4.32	12	2.68 (2.17–3.31)

Source: [19]

Comparisons of crude and birth weight-specific neonatal mortality rates show a similar pattern when ranks of countries are based on crude rates vs rates from live births with a birth weight ≥1000 g (or a gestation age ≥28 weeks). In the previously mentioned study [19], Sweden ranked first with a crude neonatal mortality rate that was half the same rate in the USA (RR 0.47, 95% CI 0.41–0.54). However, the neonatal mortality rates among live births ≥1000-g birth weight were not significantly different between Sweden and the USA (RR 0.97, 95% CI 0.83–1.14; Table 1).

As mentioned, differences in rankings based on crude versus such birth weight-specific rates of stillbirth and neonatal death reflect international differences in the registration of births at the borderline of viability. In fact, the potential for such bias in international comparisons of fetal and infant mortality rates due to differences in birth registration has been recognized for decades and the World Health Organization (WHO) recommends that international comparisons of infant mortality be restricted to live births with a birth weight ≥1000 g [20, 21].

## International Variation in Birth Registration Criteria

The longstanding WHO definition of live birth includes all births showing any evidence of life, irrespective of birth weight or gestational age [20, 21]. On the other hand, WHO has proposed a singular viability criterion for the national reporting of perinatal mortality rates, viz., a birth weight ≥500 g [20, 21]. If birth weight is unavailable, the WHO recommends the use of a ≥22-week gestation criterion for stillbirth reporting, and if that information is also missing, a crown–heel length of ≥25 cm [20, 21]. Table 2 lists the birth registration criteria in selected high-income countries and shows the substantial international variation in stated criteria [22, 23]. Of particular note is the use of dual birth weight and gestational age criteria for stillbirth registration in many countries, which contrast with the WHO-recommended singular birth weight criterion. Use of dual criteria, for example, ≥22 weeks’ gestation *or* ≥500-g birth weight, means that the gestational age criterion will restrict registration to stillbirths with a gestational age of ≥22 weeks, whereas the birth weight criterion will require the registration of variable proportions of stillbirths delivered at 19, 20, 21, 22, 23, and 24 weeks’ gestation (since 500 g represents the median birth weight of births at 22 weeks’ gestation [24]). Also notable is the disconnection between the birth weight and gestational age criteria in some countries. Although Australia and Canada both have an identical gestational age criterion (i.e., ≥20 weeks), Canada has an additional birth weight criterion (i.e., ≥20 gestational age or ≥500-g birth weight). The Canadian criteria in particular and the dual criteria of other countries in general reveal a lack of conceptual clarity regarding how viability is to be standardized.

**Table 2:** Birth weight, gestational age, and/or other criteria for stillbirth and live birth registration in selected high-income countries

Country	Stillbirth	Live birth	Termination of pregnancy
Sweden	≥28 weeks	Any signs of life	Included
Finland	≥22 weeks or ≥500 g	Any signs of life	Not included
Germany	≥500 g	Any signs of life	Not included
Czech Republic	≥22 weeks	≥500 g or any weight surviving first 24 h	Not included
Estonia	≥22 weeks or ≥500 g	Any signs of life	Not included
Poland	≥500 g	≥500 g	Not included
England and Wales	Legal limit of ≥24 weeks; voluntary notification at 22-23 weeks	Any signs of life	Included
United States	≥20 weeks^a^	Any signs of life	Not included
Latvia	≥22 weeks	Any signs of life	Not included
Netherlands	≥22 weeks or ≥500 g	≥24 weeks for civil registration; ≥16 weeks for perinatal registry	Included
Canada	≥20 weeks or ≥500 g	Any signs of life	Included
Australia	≥20 weeks or ≥400 g if gestation is unavailable	Any signs of life	Included
Denmark	≥28 weeks till 2003; ≥22 weeks from 2004	Any signs of life	Not included

Source: [22–24]

^a^Gestational age 20 weeks or greater in 25 states, 20 weeks of gestation or greater or 350-g or greater birth weight in 12 states, and various other definitions used in the remaining states [25, 26]

## Birth Registration Criteria Versus Birth Registration Practices

International variation in criteria for birth registration is stark especially with regard to stillbirth, and this precludes comparisons of crude perinatal mortality rates by country. However, there are factors beyond the stated differences in birth registration criteria that further affect registration practices including financial compensation for care providers (for identifying a pregnancy outcome as a spontaneous miscarriage, a live birth, or a stillbirth), health care culture (regarding live birth registration if the probability of survival is non-existent or extremely low), and the method of gestational age ascertainment.

Evidence for these assertions is seen in variations in stillbirth-to-live birth ratios among births at the borderline of viability within different states in the USA [25]. Further support for this proposition is provided by neonatal mortality rates at extremely preterm gestation in different countries [27••]. Table 3 shows the gestational age distribution of live births and gestational age-specific neonatal mortality in Canada and Sweden for the period 1995 to 2005 (both countries register all live births irrespective of gestational age or birth weight). Although live birth rates at 24–25, 26–27, and 28–31 weeks were similar between Canada and Sweden in 1995–2005, the frequencies of live births <22 weeks were not reported in Sweden and live births at 22–23 weeks were significantly more frequent in Canada compared with Sweden [27••]. Also, among live births at 22–23 weeks’ gestation, the neonatal mortality rate in Canada (892.2 per 1000 live births) was substantially and significantly higher than the neonatal mortality rate in Sweden (561.2 per 100 live births). The unexpectedly low neonatal mortality rate observed in Sweden at 22–23 weeks’ gestation in 1995–2005 is likely the result of selective birth registration of survivors and subsets of extremely preterm infants with a relatively good prognosis. Although the mortality differential between Canada and Sweden at 22–23 weeks’ gestation (and also at 24–25 weeks) could have occurred because of differences in the quality of neonatal care, the rates of death in the two countries at 26–27 and 28 weeks and beyond [27••] belie this explanation. Despite increases in the survival of infants born at 22–23 weeks’ gestation in recent decades, mortality rates remain substantial; research studies show that hospitals of the National Institute of Child Health and Human Development Neonatal Research Network in the USA recorded neonatal mortality rates of 948 per 1000 live births (95% CI 917–968) at 22 weeks and 691 per 1000 live births at 23 weeks (95% CI 654–725) in 2008–2011 [28].

**Table 3:** Gestational age distribution and gestational age-specific neonatal mortality rates in Canada and Sweden, 1995–2005

Gestational age (weeks)	Canada 1995–2005			Sweden 1995–2005		
	Live births		Neonatal mortality per 1000 (95% CI)	Live births		Neonatal mortality per 1000 (95% CI)
	Number	Rate/100		Number	Rate/100	
<22	1494	0.07	950.5 (938.2–960.9)	Not reported	–	–
22–23	1902	0.08	892.2 (877.4–905.8)	335	0.03	561.2 (506.2–615.1)
24–25	2774	0.12	417.4 (399.0–436.1)	1056	0.10	269.9 (243.3–297.8)
26–27	3982	0.17	141.9 (131.2–153.1)	1633	0.16	140.2 (123.7–158.0)
28–31	14,820	0.65	44.4 (41.1–47.8)	6628	0.64	42.2 (37.5–47.4)
32–36	146,954	6.40	7.0 (6.6–7.4)	55,058	5.35	8.6 (7.8–9.4)
37–41	2,093,277	91.1	0.9 (0.9–0.9)	882,169	85.8	0.8 (0.7–0.9)
≥42	32,197	1.40	1.3 (0.9–1.7)	81,697	7.94	1.2 (1.0–1.5)
Unknown	5699	0.25	32.3 (27.9–37.2)	1273	0.12	33.8 (24.6–45.2)
≥22	2,295,906	99.9	3.1 (3.1–3.2)	1,028,576	100.0	2.2 (2.1–2.3)
≥24	2,294,004	99.8	2.4 (2.3–2.5)	1,028,241	100.0	2.4 (2.2–2.7)
≥28	2,287,248	99.6	1.7 (1.6–1.7)	1,025,552	99.7	1.6 (1.4–1.7)
Total	2,303,099	100.0	3.8 (3.7–3.8)	1,029,849	100.0	2.2 (2.1–2.3)

Source: [27••]

## International Variation in Stillbirth Definitions

Table 2 shows the differences in criteria for stillbirth registration in selected high-income countries. Beyond differences in birth weight and gestational age criteria, there are crucial differences with regard to the registration of pregnancy terminations (i.e., medical and surgical abortions). Many countries such as Finland, Germany, and the USA exclude pregnancy terminations from stillbirth counts, whereas other countries such as Australia, Canada, England and Wales, and Sweden include pregnancy terminations if birth weight and gestational age criteria for stillbirth registration are satisfied [22–24]. Inclusion of pregnancy terminations in stillbirth counts can result in drastically higher stillbirth rates, especially in countries with a lower gestational age criterion for stillbirth registration. Thus, countries such as Australia, Canada, and New Zealand, which register pregnancy terminations at ≥20 weeks’ gestation as stillbirths, are more affected than other countries, such as England and Wales and Sweden, which also register pregnancy terminations as stillbirths but have a higher gestational age cutoff for registration (≥24 and ≥28 weeks’ gestation, respectively).

Contemporary stillbirth registration is based on gestational age at birth or birth weight rather than gestational age or weight at the time of fetal death. This leads to some unexpected birth registration conundrums especially because of new technologies such as fetal reduction for multifetal pregnancy [23, 24]. Fetal reduction is a procedure carried out at 10–12 weeks’ gestation in order to improve outcomes of multifetal pregnancy, especially higher-order multifetal pregnancy. Thus, a fetal reduction procedure could be used to reduce a triplet pregnancy to a twin (or singleton) pregnancy as the latter is less likely to result in preterm birth and associated complications such as neonatal mortality and severe neonatal morbidity. However, the fetus(es) reduced at 10 weeks’ gestation and delivered along with healthy twins (or a healthy singleton) at late preterm or term gestation requires stillbirth registration (since stillbirths are defined on the basis of the gestational age when the stillborn fetus is delivered and not when the fetal death occurred). There is a legal requirement for such stillbirths to be registered in countries such as Canada [23, 24], while more pragmatic procedures are followed in other countries. Not surprisingly, increases in pregnancy terminations following the prenatal diagnosis of serious congenital malformations and fetal reductions have resulted in steady increases in stillbirth rates in Canada in recent decades [29]. Standardization of birth registration criteria including those related to viability and uniformity with regard to inclusion/exclusion of pregnancy terminations from stillbirth counts are required in order to address current biases that invalidate international comparisons of perinatal mortality.

## International Comparisons Based on Very Preterm Infants from Neonatal Networks

Several neonatal networks have been created in recent years, with neonatologists championing the collection of data from neonatal intensive care units for quality assurance and research purposes. The development of databases documenting the experience of very preterm infants has facilitated comparisons of neonatal mortality and severe morbidity between centers and also between countries [4–7]. For instance, a recent study compared neonatal mortality and severe morbidity rates among very preterm and very low-birth-weight infants in Canada, Israel, Japan, Spain, Sweden, Switzerland, and Taiwan [5]. The methodology of such studies typically involves contrasts of between-country rates of neonatal mortality or composite neonatal mortality and severe morbidity with logistic regression adjustment for extraneous determinants such as gestational age, birth weight for gestational age (*z* score), multiple birth, infant sex, and antenatal corticosteroid use.

Such neonatal intensive care unit network databases have facilitated the conduct of epidemiologic studies examining the effects of maternal characteristics and interventions among very preterm or very low-birth-weight infants. However, some of the studies based on very preterm or very low-birth-weight subpopulations have shown counterintuitive findings. For instance, one such study [30] showed that very preterm infants of mothers with hypertensive disorders of pregnancy had a reduced risk of neonatal death compared with the very preterm infants of mothers without hypertension (adjusted odds ratio 0.77, 95% CI 0.67 to 0.88). Another study [31] showed that neonatal mortality among very preterm infants decreased with increasing maternal age. The latter study showed significantly different neonatal mortality rates of 9.0, 7.4, 6.5, 6.5, and 6.1% among infants of mothers aged 21–25, 26–30, 31–35, 36–40, and 41–54 years, respectively [31]. The mortality advantage conferred by older maternal age remained significant after adjustment for gestational age at birth, use of prenatal steroids, multiple births, prenatal care, chorioamnionitis, and cesarean delivery. On the other hand, comparisons of mortality rates between the very preterm infants of mothers who smoked vs very preterm infants of non-smoking mothers showed no difference overall [32]. In fact, stratified analyses showed non-significantly higher mortality rates among infants of smokers at 26–28 weeks’ gestation and significantly higher mortality rates among infants of smokers at 29–32 weeks’ gestation.

## Problems with International Comparisons of Mortality Among Very Preterm Infants

Over four decades ago, Yerushalmy showed that neonatal mortality rates among low-birth-weight infants of mothers who smoked were significantly lower than the neonatal mortality rates among low-birth-weight infants of mothers who did not smoke [33]. This association was reversed at birth weights above approximately 3000 g, with neonatal mortality rates among the larger infants of mothers who smoked being higher than those among the larger infants of non-smoking mothers. As well, unstratified comparisons (i.e., among all infants irrespective of birth weight) showed that neonatal mortality rates were relatively higher among infants of mothers who smoked. Subsequent studies have confirmed these findings and also shown this to be a general phenomenon that is also observed in contrasts by plurality (singletons vs twins), race (blacks vs whites), parity (nulliparous vs parous women), maternal age (older vs younger mothers), pregnancy complications (women with hypertension in pregnancy vs normotensive women), etc. [8–18, 34–37]. Such contrasts also show a mortality crossover across early versus later gestation and with regard to stillbirth [8, 12, 15–17, 35–37], and the phenomenon has been labeled the paradox of intersecting perinatal mortality curves.

Several solutions have been proposed as explanations for this paradox including one based on the fetuses-at-risk approach, which involves a simple reformulation of denominators for calculating gestational age-specific stillbirth and neonatal death rates. Under this fetuses-at-risk formulation, gestational age-specific stillbirth and gestational age-specific neonatal mortality rates are calculated not based on total births or live births at a particular gestation but on the fetuses at risk for perinatal death at that gestation [17, 18]. This formulation resolves the paradox of intersecting perinatal mortality curves and shows that infants of mothers with adverse influences such as maternal smoking [35], hypertension [37], multifetal pregnancy [17], older maternal age [38], and race [35], have relatively higher mortality compared with infants of mothers without the said adverse influence [39–41]. Advocates of the fetuses-at-risk formulation argue that this model, which treats gestational age as survival time, provides causal insights into the etiologic role of risk factors such as maternal smoking, hypertensive disorders of pregnancy, and multifetal pregnancy [39]. On the other hand, the traditional perinatal formulation (under which births at a particular gestation are used in the denominator for perinatal mortality rates) works as a non-causal prognostic model [39]: a mother with preeclampsia who delivers a 2000-g baby can be reassured post hoc that the baby’s prognosis is relatively favorable (but not that pre-eclampsia causes better perinatal outcomes).

Other explanations for the paradoxical perinatal mortality crossover include the relative birth weight hypothesis which postulates that each subpopulation (singletons, twins, etc.) has a mortality pattern related to its birth weight (or gestational age) distribution [9]. Contrasts of mortality based on relative birth weight or relative gestational age (i.e., expressed in standard deviation units above or below the mean birth weight or gestational age) will not exhibit the paradox of intersecting perinatal mortality curves. Relative birth weight- and relative gestational age-specific perinatal mortality contrasts show higher death rates among infants of mothers who smoke (have hypertension, multifetal pregnancies, etc.) at all birth weights and all gestational ages [9]. A third explanation for the intersecting perinatal mortality paradox, termed the collider stratification bias, proposes uncontrolled confounding of the relation between birth weight (or gestational age) and perinatal mortality as the cause for the mortality crossover [42, 43]. Although there is little consensus on the preferred solution for the paradox of intersecting perinatal mortality curves [44••, 45–47, 48••, 49], there is general consensus that maternal smoking, hypertensive disorders, and multifetal pregnancy lead to adverse fetal and neonatal outcomes at all gestational ages and birth weights.

**Fig. 1:**
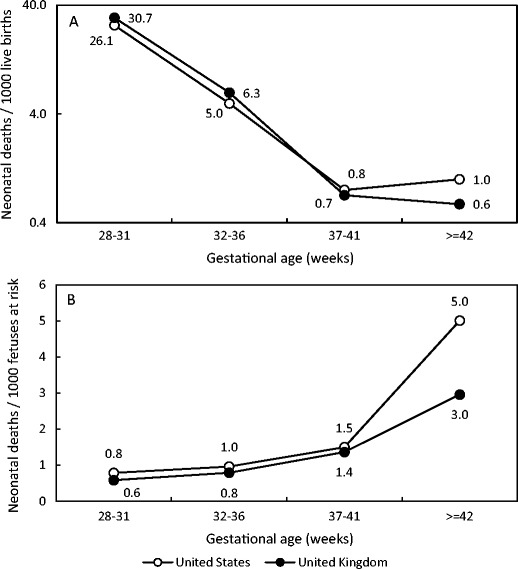
Gestational age-specific neonatal mortality rates in the USA (2013) and in the UK (2014). **a** Rates expressed using the traditional formulation (per 1000 live births at each gestational age). **b** Rates expressed under the fetuses-at-risk formulation (per 1000 fetuses at risk of neonatal death at that gestational age, corrected for duration of the gestational period). Source: Centers for Disease Control and Prevention Wonder Online Database and the MBRRACE-UK Perinatal Mortality Surveillance Report 2016 [48••]

International comparisons of the neonatal mortality among very preterm and very low-birth-weight infants are as seriously flawed as comparisons of neonatal mortality by maternal smoking, hypertensive disorders, and multifetal pregnancy, which are restricted to the preterm subpopulation. The problem with international comparisons of neonatal mortality restricted to the very preterm birth range is illustrated in Fig. 1, which contrasts the neonatal mortality rate ≥28 weeks in the USA vs the neonatal mortality rate ≥28 weeks in the UK in 2013–2014 [50]. Whereas the neonatal mortality rate ≥28 weeks in the USA was significantly higher (1.5, 95% CI 1.5–1.5 per 1000 live births) than the neonatal mortality rate ≥28 weeks in the UK (1.3, 95% CI 1.2–1.4 per 1000 live births), the traditional estimate of gestational age-specific neonatal mortality showed that neonatal mortality rates at 28–31 and 32–36 weeks’ gestation were significantly lower in the USA compared with the same rates in the UK (Fig. 1a). The opposite was true at term and postterm gestation. Thus, a between-country comparison of neonatal mortality restricted to infants at 28–31 weeks would erroneously conclude that the USA had a lower mortality than the UK. On the other hand, the same gestational age-specific neonatal mortality rates, calculated under the fetuses-at-risk formulation, show higher neonatal mortality in the USA in each gestational age subgroup (Fig. 1b).

## Birth Weight and Gestational Age-Specific Comparisons of Stillbirth and Neonatal Mortality

The combination of a lack of international standardization with regard to birth registration procedures and practices and the methodologic problems inherent in the traditional model of birth weight- and gestational age-specific perinatal mortality mean that comparisons of perinatal mortality rates between countries can only be valid at a gestational age beyond which complete registration occurs and based on the fetuses-at-risk formulation. The latter approach requires that gestational age-specific perinatal mortality comparisons be made at gestational ages ≥24, ≥26, or ≥28 weeks’ gestation, etc., depending on the gestational age at which complete birth registration is assured.

Although restricting the population to a gestational age above which complete registration is assured restores validity to international comparisons of perinatal mortality, it results in the exclusion of a substantial fraction of the population. For instance, in countries such as Canada, over 40% of infant deaths occur among live births <28 weeks’ gestation (birth weight <1000 g) and an international comparison of live births ≥28 weeks’ gestation will exclude this vulnerable population [51]. The enormous costs that accrue annually for the care of such extremely low-birth-weight and preterm infants show the importance accorded to this subpopulation, and their exclusion from international comparisons merely reflects a methodologic tactic that avoids the bias introduced by international variation in birth registration.

## Adjustment for Extraneous Characteristics

Routine reports of the UNICEF, World Bank, and OECD include comparisons of crude fetal and infant mortality without adjustment for extraneous characteristics such as maternal age and parity. If international comparisons of perinatal mortality are motivated by a desire to evaluate perinatal health status and perinatal health services, adjustment for maternal age, parity, and other maternal characteristics would be warranted. On the other hand, the neonatal networks making international comparisons of neonatal mortality among very preterm infants adjust for various extraneous determinants including receipt of antenatal corticosteroids and mode of delivery [5]. Presumably, the latter adjustment is motivated by a desire to isolate the effects of neonatal care and exclude international differences due to variations in obstetric practice.

If the motivation behind international comparisons of perinatal mortality is to assess the relative quality of health services, adjustment for obstetric, neonatal, and other health services would be contraindicated, especially if such services were designed to reduce perinatal mortality. In fact, antenatal corticosteroid administration and mode of delivery lie in the causal pathway between the health care system performance and perinatal mortality. One extraneous health service determinant of perinatal mortality, namely, use of assisted reproduction, presents a conceptual challenge in such analyses—should international comparisons of perinatal mortality adjust for use of assisted reproduction? This technology, which enables infertile couples to achieve their reproductive choices, may require adjustment as it is a marker/risk factor for perinatal death and yet distinct in purpose from other perinatal health care services.

## Miscellaneous Considerations

Although gestational age considerations are vitally important in ensuring standardized international comparisons of perinatal mortality, there are likely differences in the modality of gestational age ascertainment by country. In most high-income countries, early ultrasound dating is used to resolve problems due to inaccurate gestational age estimates based on menstrual dates. However, not all women receive early ultrasonography, the accuracy of such measurement decreases with advancing gestation, and the proportion of women receiving early ultrasonography likely varies by country. Additionally, there are significant differences in how ultrasonographic measurements are made, and between- and even within-observer variation in measurements is not inconsequential [52]. There is no standard international algorithm for determining gestational age that combines menstrual dating, ultrasonographic measurements, pediatric examination, and other modalities of gestational age ascertainment.

A final consideration in international comparisons of perinatal mortality relates to prenatal diagnosis and pregnancy termination for serious congenital malformations. Although the gestational age at which pregnancy termination is carried out following prenatal diagnosis of a serious congenital anomaly is falling rapidly, a significant fraction of such cases occur in the 20–23 weeks’ gestational age range [29]. Excluding stillbirths that follow prenatal diagnosis and pregnancy termination from international comparisons of perinatal mortality may be defensible insofar as such procedures reduce parental anguish and societal costs associated with congenital anomalies that will likely result in late fetal or infant death. On the other hand, a counterargument could be made that deaths following prenatal diagnosis and pregnancy termination fulfill birth registration criteria for stillbirth and can be viewed as the result of procedures that merely shift the timing of death. It is noteworthy that WHO recommendations regarding international comparisons of infant mortality (which preceded widespread uptake of prenatal diagnosis and pregnancy termination for serious malformations) advocate comparisons of infant mortality excluding deaths due to congenital anomalies [21].

## Conclusions

Differences in birth registration between countries include differences in birth weight and gestational age criteria and also in the requirement to include or exclude pregnancy terminations (that satisfy birth registration requirements) from stillbirth counts. These differences in birth registration compromise international comparisons of crude perinatal mortality rates. International comparisons of perinatal mortality restricted to births above a specific birth weight or gestational age (i.e., that are unaffected by birth registration differences) yield rankings of perinatal mortality that are substantially different from ranks based on crude comparisons. Although gestational age- and birth weight-specific contrasts of births ≥28 weeks’ gestation or ≥1000-g birth weight yield valid results, they suffer the drawback of excluding a substantial group of vulnerable infants from international comparisons. Comparisons of neonatal mortality among very preterm and very low-birth-weight infants, which are based on information from national neonatal networks, are also compromised because rates are based on incomplete denominators. Use of very preterm or very low-birth-weight live births as the denominator leads to the paradox of intersecting perinatal mortality curves and potentially biased results. Although not universally accepted, the fetuses-at-risk approach addresses this problem but requires a change in denominators to the fetuses at risk of perinatal death at each gestation.

In summary, international comparisons of crude perinatal mortality rates, while laudable as a means for spurring improvements in national health care systems, remain compromised currently. Remedial action will require both improved standardization with regard to birth registration issues and also conceptual progress with regard to identifying appropriate denominators for calculating gestational age-specific perinatal mortality rates.
